# Supplementary material for: Diel cycle of sea spray aerosol concentration
Source: Nat Commun. 2021 Sep 16;12:5476. doi: 10.1038/s41467-021-25579-3 (PMC8445914; doi:10.1038/s41467-021-25579-3)
Supplement: Supplementary file 1 — Supplementary Information [file 41467_2021_25579_MOESM1_ESM.pdf]

Supplementary Information for

Diel cycle of sea spray aerosol concentration

J. Michel Flores[1*], Guillaume Bourdin[2,3], Alexander B. Kostinski[4], Orit Altaratz[1], Guy Dagan[5], Fabien Lombard[3], Nils Haëntjens[2], Emmanuel Boss[2], Matthew B. Sullivan[6], Gabriel Gorsky[3,7], Naama Lang-Yona[8,9], Miri Trainic[1], Sarah Romac[10], Christian R. Voolstra[11], Yinon Rudich[1], Assaf Vardi[8*], and Ilan Koren[1*]

Affiliations:

[1] Weizmann Institute of Science, Department of Earth and Planetary Sciences, 7610001 Rehovot, Israel.

[2] School of Marine Sciences, University of Maine, Orono, ME 04469, USA.

[3] Sorbonne Université, CNRS, Laboratoire d'Océanographie de Villefranche, F-06230 Villefranche-sur-Mer, France.

[4] Department of Physics, Michigan Technological University, Houghton, MI 49931, U. S. A.

[5] Atmospheric, Oceanic and Planetary Physics, Department of Physics, University of Oxford, Oxford OX1 3PU, UK.

[6] Ohio State University, Departments of Microbiology and Civil, Environmental and Geodetic Engineering, Columbus, Ohio, 43210 U. S. A.

[7] Research Federation for the Study of Global Ocean Systems Ecology and Evolution, FR2022/Tara Oceans-GOSEE, Paris, France

[8] Weizmann Institute of Science, Department of Plant and Environmental Science, 7610001 Rehovot, Israel.

[9] Now at: Civil and Environmental Engineering, Technion - Israel Institute of Technology, 3200003 Haifa, Israel.

[10] Sorbonne Université, CNRS, Station Biologique de Roscoff, AD2M, UMR 7144, ECOMAP 29680 Roscoff, France.

[11] Department of Biology, University of Konstanz, 78457 Konstanz, Germany.

*Corresponding authors: flores@weizmann.ac.il, assaf.vardi@weizmann.ac.il, ilan.koren@weizmann.ac.il

This PDF file includes:

Supplementary Note 1. Effect of Tara and local influence on the diel cycle of SSA
Supplementary Figures 1 to 15
Supplementary Tables 1 to 2

Supplementary Note 1. Influence of the boat and instrumentation setup on the measured SSA concentration

To discard a possible local influence by the boat on our finding, we explored the effect of engine contamination, daily routines on board, daily heating of the instrument, or a relative humidity effect.

In Flores et al.[1] we showed that the aerosol measurements were affected by engine contamination only along the first month of the data (i.e., the Atlantic transect) when the inlets were located at ~15 m height. After this period the inlets were moved to the top of the mast. Hence, as part of this analysis the first month of data was cleaned from pollution and all the rest of the data used in this manuscript was measured after moving the inlets to the top of the mast, which rules out any interference by the boat.

Additionally, the OPC instrument was new at the beginning of the campaign and it was checked and recalibrated by the company when *Tara* anchored in the island of Guam, Micronesia (about 8 months after the beginning of the expedition), assuring a well-functioning instrument.

The OPC was installed in the aft of the boat. A special sensor measured the temperature and RH of the sampled air after the Nafion dryer. We did not see RH or temperature fluctuations at the time of change in aerosol concentration, ruling out that RH effects caused the diel changes in aerosol concentration. Moreover, the temperature in the room and after the dryer was always below 40°C, the OPC is made to function properly up to +50 °C, hence the temperature is within its predefined specifications.

Finally, we checked a possible influence of the daily routines on the boat on our measurements. The boat's route combined sailing periods with days to week-long stops near islands (the cycle was observed during the 2 types of periods). The OPC was continuously running connected to its own inlet tubbing (separated from the filter system), and was monitored from another part of the boat. The OPC was untouched unless it showed an error (and in that case, it was turned off, and fixed). Twice a day, between 8-10am and 8-10pm the filters from the custom-made aerosol filter system were changed. The exact times of the filters are provided in Supplementary Table 1. The daily changes in concentration measured by the OPC happened around 06:00 and 17:00-18:00 local time, when people on board were either asleep or doing other daily routines. We could not identify regular actions on board that could have caused the diurnal aerosol concentration changes.

Supplementary Figures

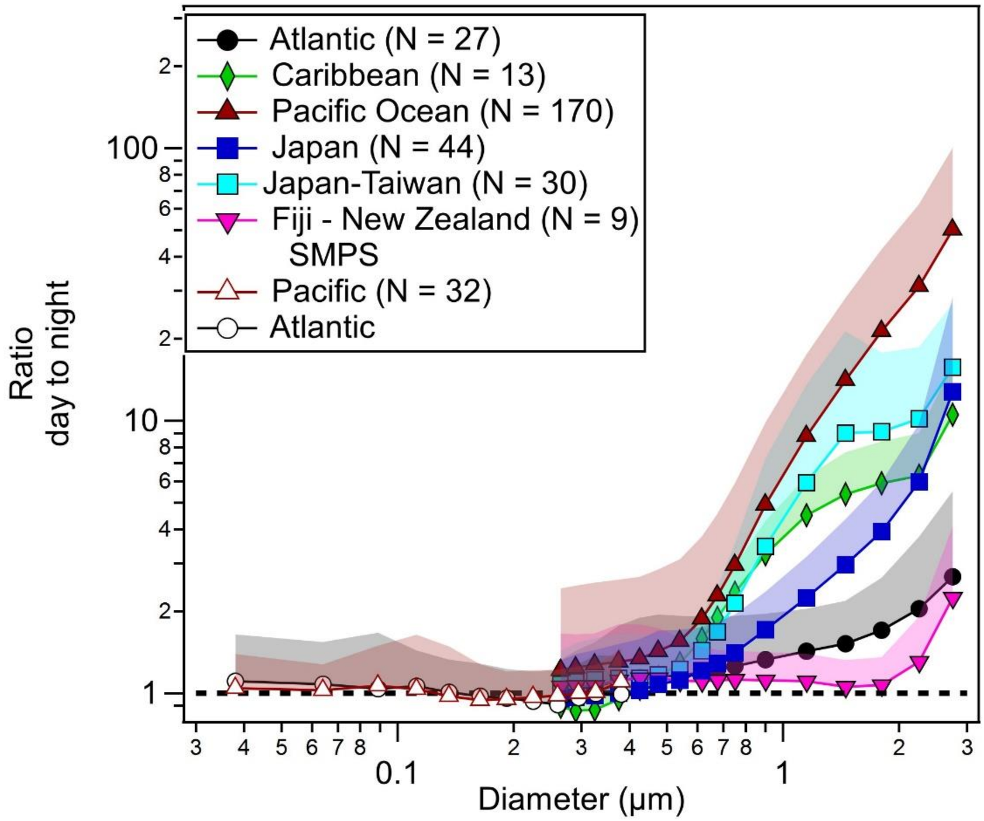

Supplementary Figure 1. Mean day-to-night aerosol count ratio for each bin for different regions across *Tara's* route. The full markers are the same as in Fig. 2 of the main text. The open markers show two legs where we calculated the DNR for D < 0.25μm using SMPS data. The Pacific leg refers to the leg between Keelung and Fiji in the western Pacific. For clarity, the SMPS data was binned into 25 nm segments and then the DNR was calculated. The shaded areas represent 1σ, and only the top parts are shown for clarity. 'N' refers to the number of days analyzed.

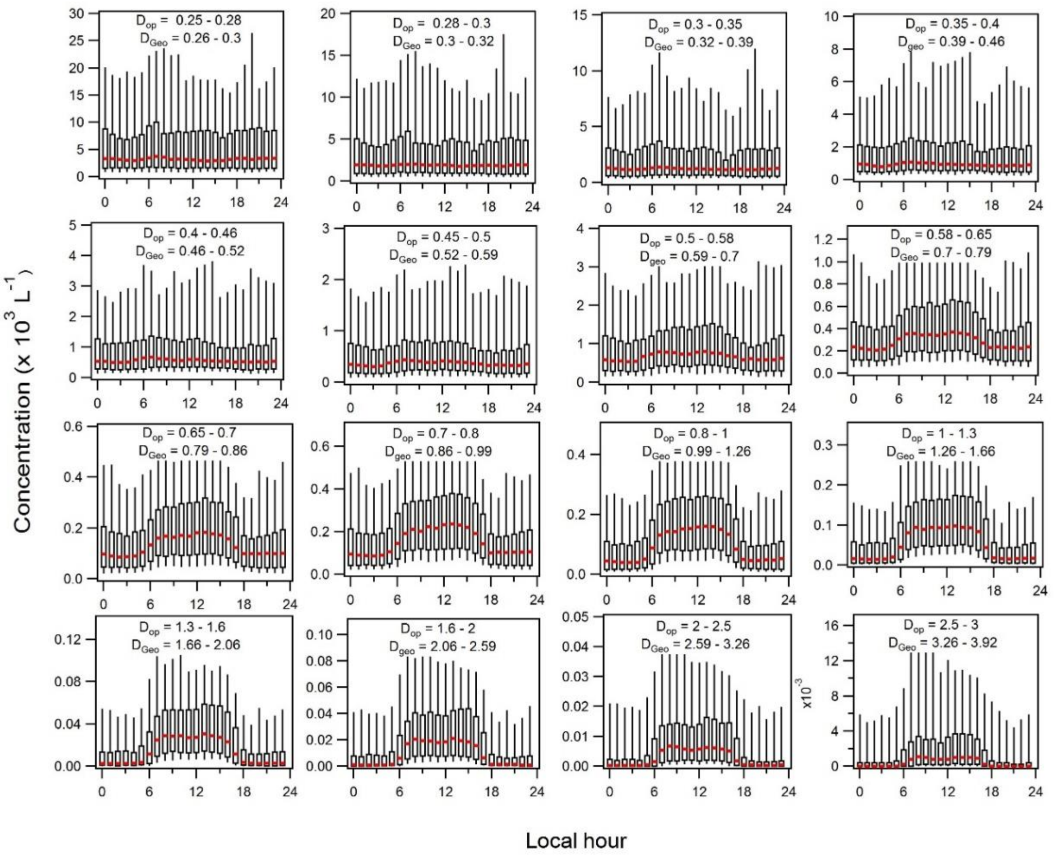

Supplementary Figure 2. Diurnal box plot analysis of total concentration of aerosol diameters per hour for 16 bins of the OPC. The box plot analysis shows the median, and the 5th, 25th, 75th, and 95th percentiles. Only data from the Pacific Ocean and when *Tara* was at least 100 km away from land were used for this analysis, in total N = 124 days (~2978 hours) were analyzed. At the top of each panel the lower and upper limit in micrometers of the OPC bins are shown. We also show the approximate geometrical diameter (D_{geo}) using a linear fit to the data shown in the Fig. 4a from Flores et al. 2009[2] for dry NaCl: $D_{Geo} = \frac{(D_{op} - 57.35)}{0.75}$. Note that the linear fit is using data only between 0.4 – 0.8μm.

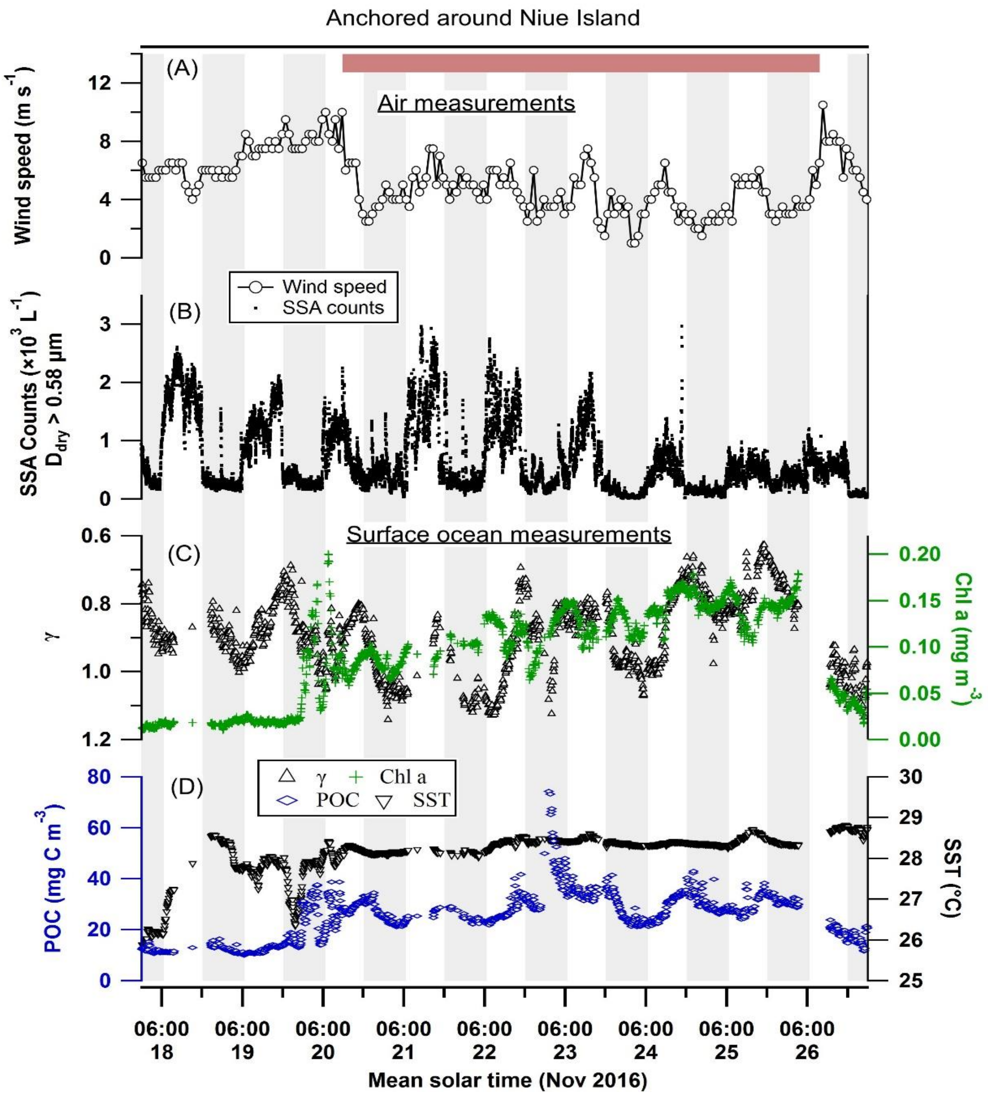

Supplementary Figure 3. Diurnal emission of SSA$_{0.58\mu m}$ when *Tara* was anchored around Niue Island. A) Wind speed measured at the top of *Tara's* mast 30 m above sea level. The Photosynthetically active radiation (PAR) measured on board *Tara* was not available in this stop over. B) Total counts per litter of aerosols with D > 0.58µm. C) Spectral exponent of the particulate beam attenuation (*γ*; inverse axis) and chlorophyll *a* concentration D) Particulate organic carbon and sea surface temperature. The red bar marks the period *Tara* was anchored.

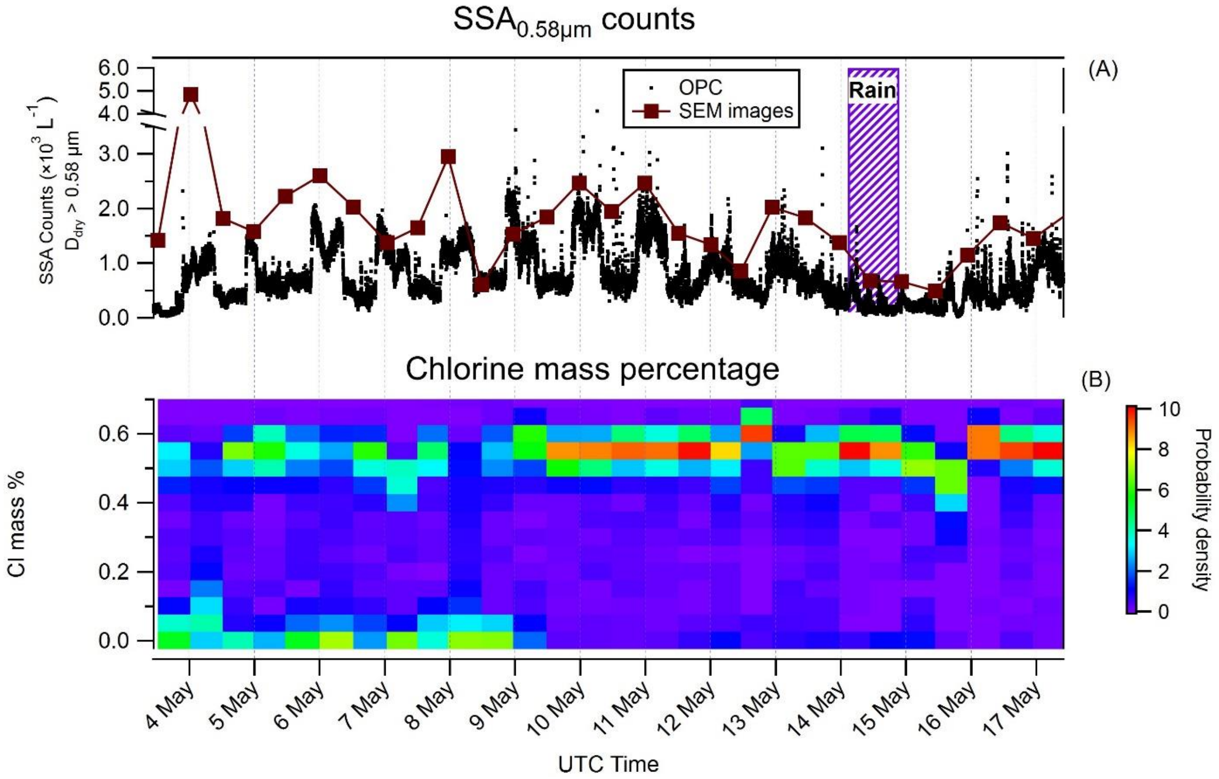

Supplementary Figure 4. Calculated SSA counts from SEM images and histograms of the Chlorine mass percentage found in each filter (normalized to probability density). The red squares show the SSA counts calculated using the SEM images and are overlaid on the SSA measurements from the OPC (black dots).

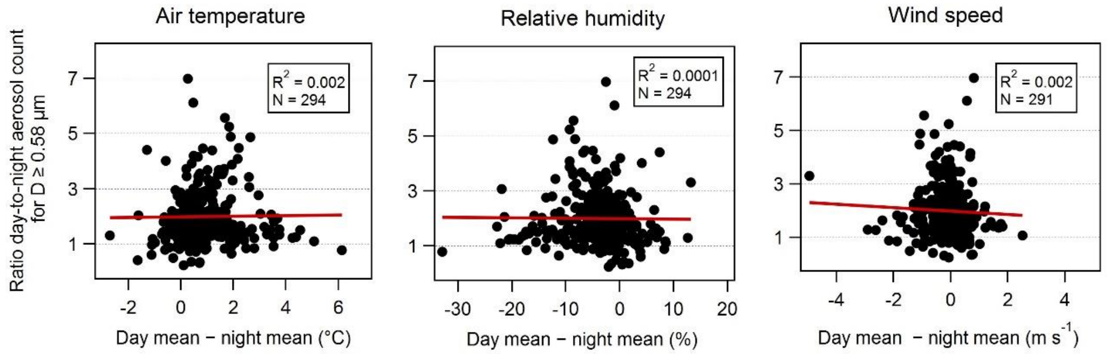

Supplementary Figure 5. Ratio of the day-to-nighttime concentration for aerosols with D ≥ 0.58µm vs the mean day-to-nighttime difference of air temperature, relative humidity and wind speed. The DNR for the atmospheric variables were calculated using the same daytime (from 07:00 to 17:00 MST) and nighttime (19:00 to 05:00 MST) periods. The red line is the linear fit to each data set. 'N' refers to the number of days analyzed. No correlation was found with any of the three atmospheric variables.

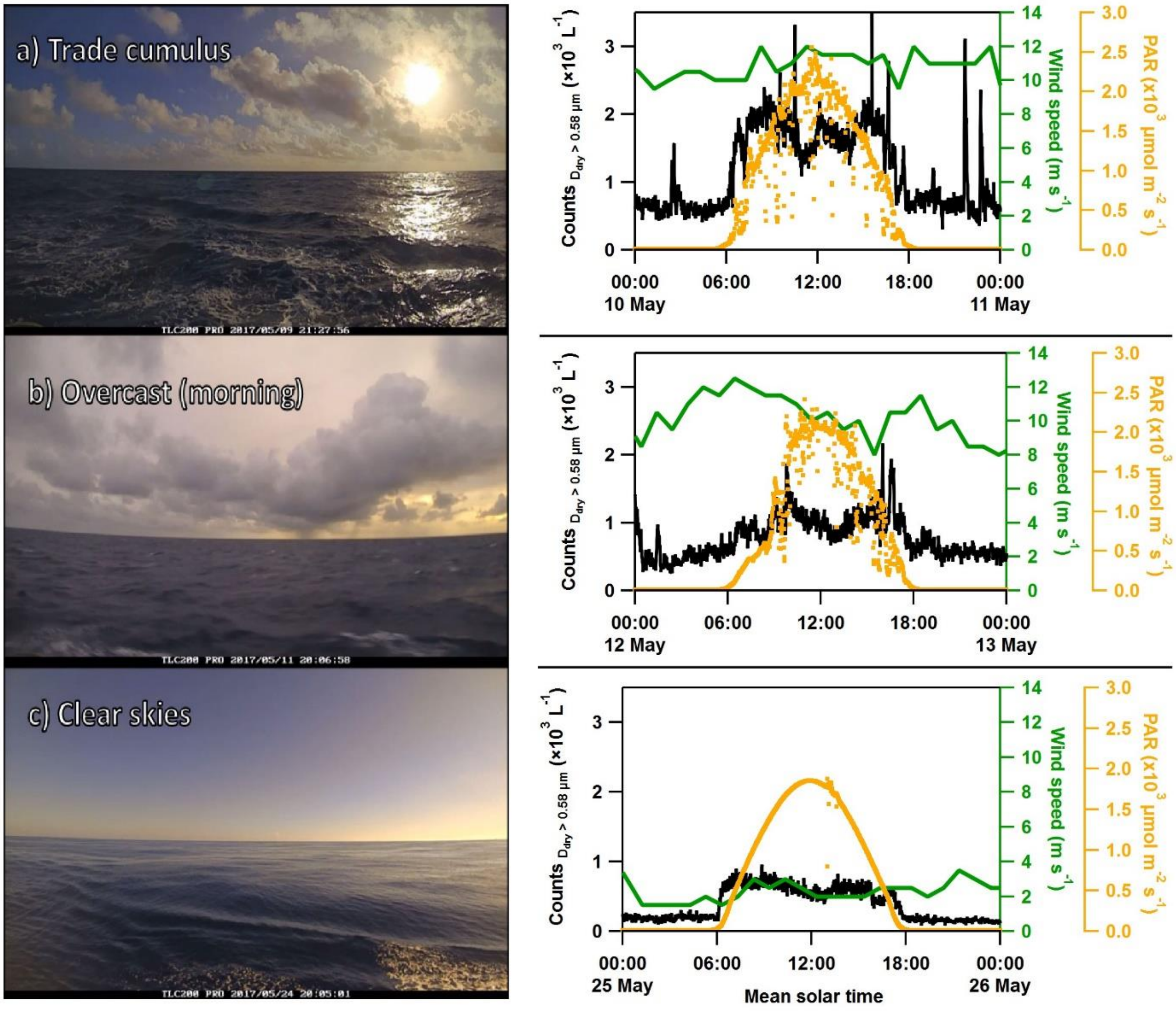

Supplementary Figure 6. Representative pictures of the ocean and atmosphere state during three distinct days. Each snapshot was taken from time-lapse videos taken during the Keelung – Fiji leg on May 2017. On the right side of each picture, the corresponding total counts for D > 0.58 µm, wind speed, and photosynthetically available radiation (PAR) measured on Tara, are shown.

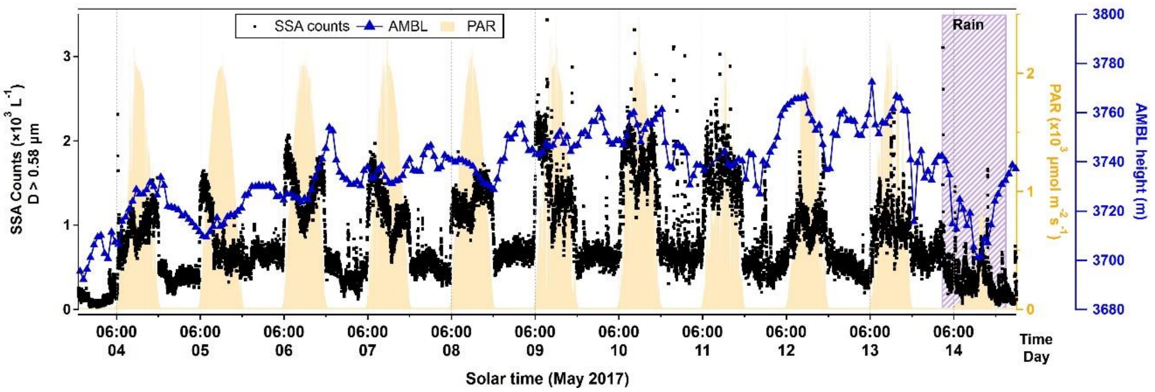

Supplementary Figure 7. Aerosol concentration per litre (D_{dry} > 0.58 µm) and the atmospheric marine boundary layer (AMBL) height from ERA5 superimposed on the photo-synthetically active radiation (PAR). The data shown here is from the orange and blue-shaded transect in the western Pacific between Keelung and Fiji (next to the double ended arrow) shown in Fig. 3 of the main text.

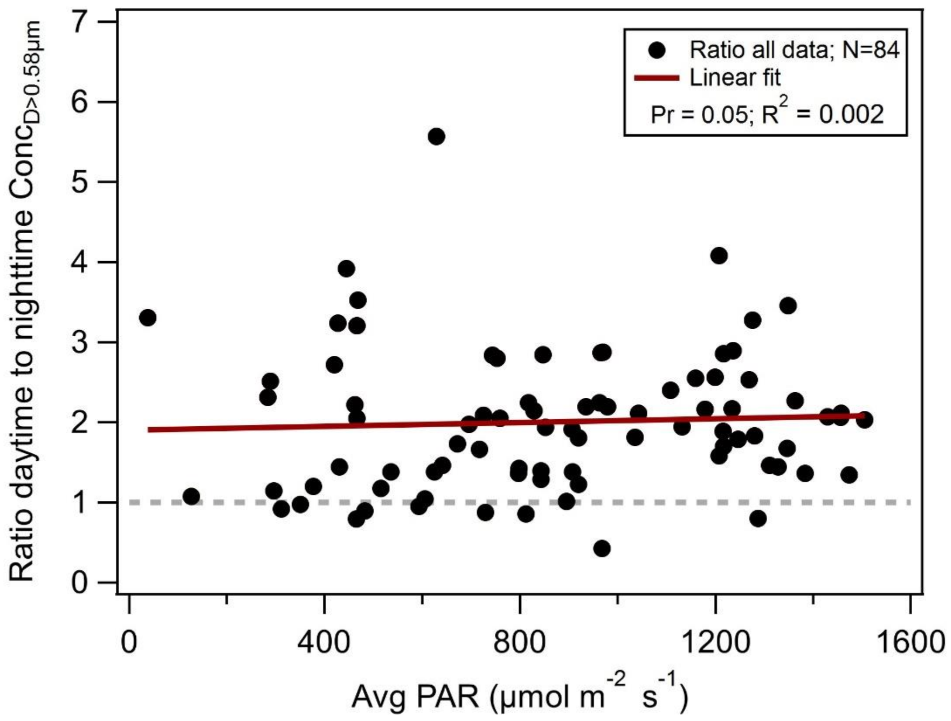

Supplementary Figure 8. Ratio of the daytime to nighttime concentration for aerosols with D > 0.58μm vs the average photosynthetically active radiation. The red line is the linear fit to all the available data. Pr refers to the Pearson correlation coefficient. ‘N’ refers to the number of days analyzed.

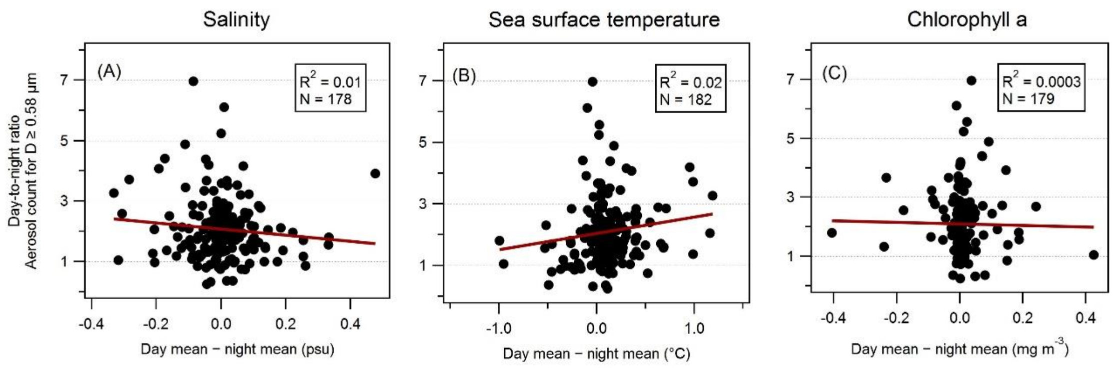

Supplementary Figure 9. Ratio of the day-to-nighttime concentration for aerosols with D > 0.58μm vs mean day-to-nighttime difference of salinity, sea surface temperature, and chlorophyll a at a depth of around 0.5 – 3.0 m. The red line is the linear fit to all the available data. 'N' refers to the number of days analyzed.

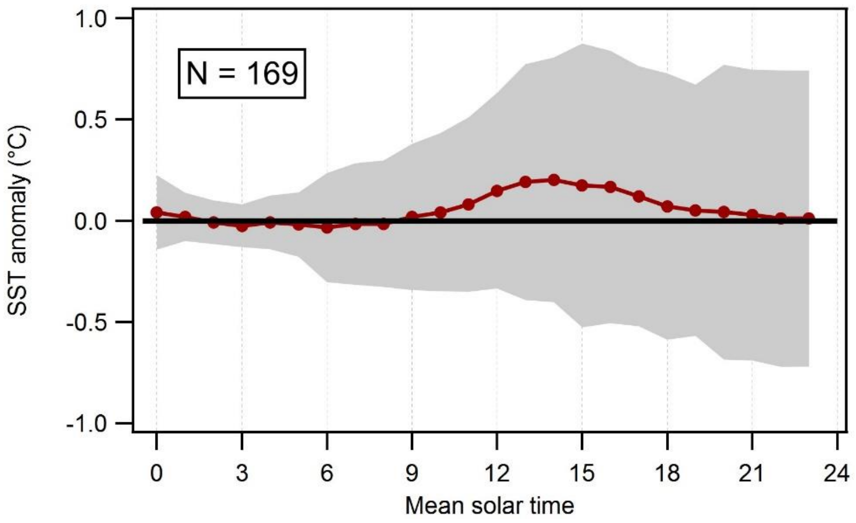

Supplementary Figure 10. Diurnal anomaly of sea surface temperature anomaly per day at a depth of around 0.5 – 3.0 m. The baseline was taken to be the mean value between midnight and five in the morning. The shaded areas are 1σ. 'N' refers to the number of days analyzed.

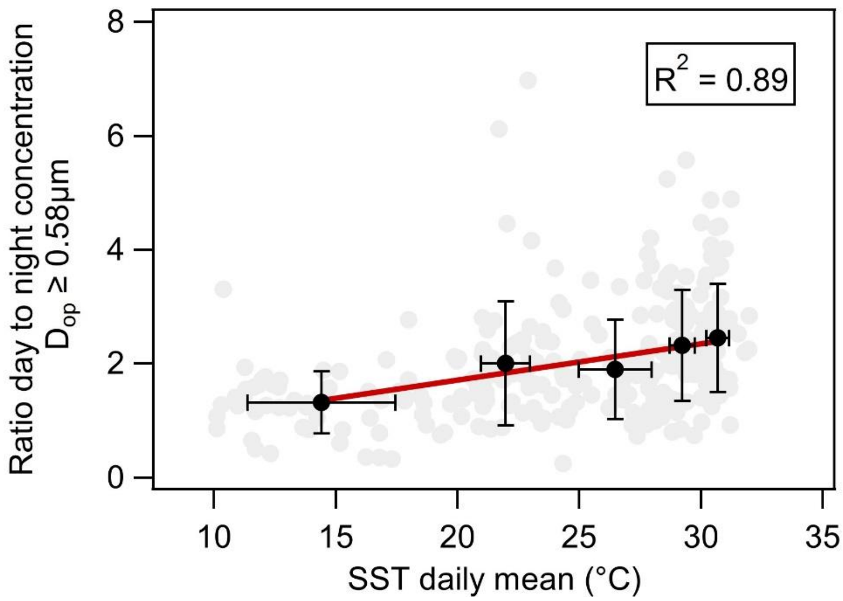

Supplementary Figure 11. Ratio of the daytime to nighttime concentration for aerosols with D > 0.58µm vs the daily mean sea surface temperature at a depth of around 0.5 – 3.0 m. The grey circles show all the data. The black circles show the mean of the data, binned into equally number of points bins (N=62 per bin). The error bars are 1σ. The red line is the linear fit to the mean data.

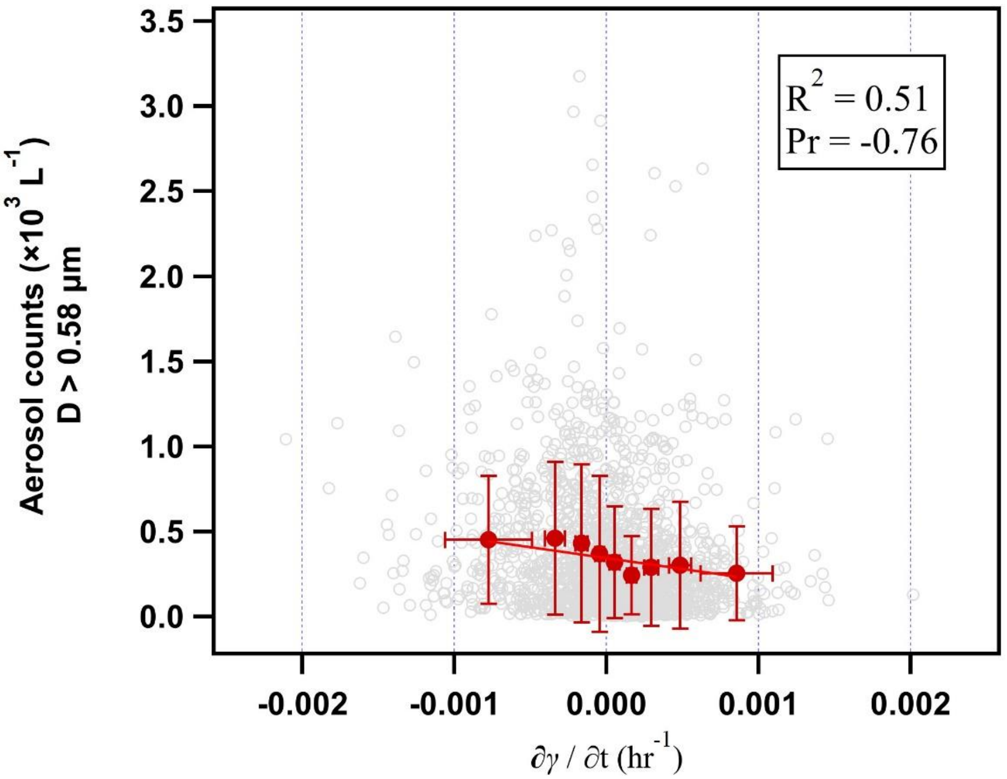

Supplementary Figure 12. $N_{SSA\geq0.58\mu m}$(h^{-1}) vs. $\partial\gamma/\partial t$ (h^{-1}) for the Pacific Ocean data. The grey circles show all the data. The red circles show the mean of the data, binned into equally number of points bins (N=223 per bin). The error bars are 1σ. The red line is the linear fit to the mean data. Pr refers to the Pearson correlation coefficient.

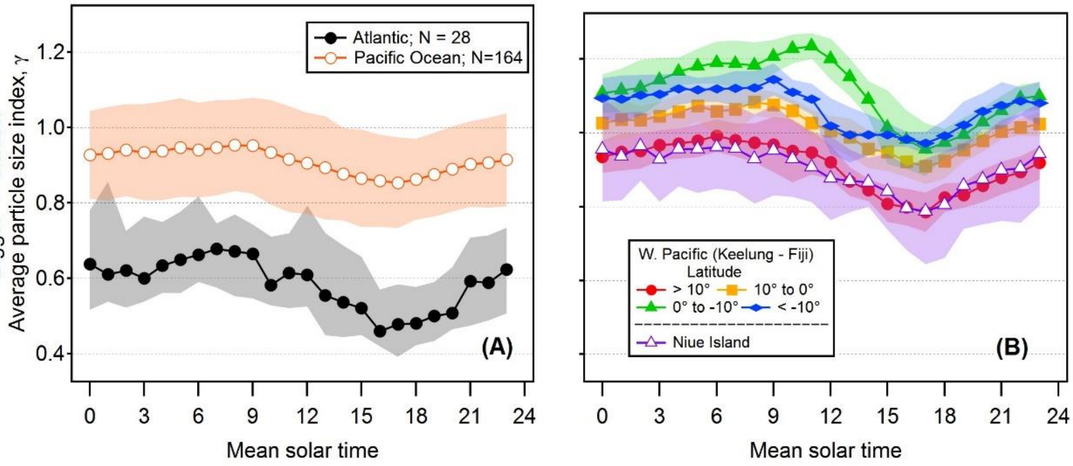

Supplementary Figure 13. Mean particle size index, *γ*, in different oceanic regions. **(A)** In the Atlantic   were measured (open orange circles). (**B)** During the Keelung – Fiji transect for latitudes above 10°N (red circles; N=10), between the Equator and 10°N (orange squares; N=9), between the Equator and 10°S (green triangles; N=8), and below 10°S (blue diamonds; N=4). The mean *γ* values while *Tara* was anchored near Niue Island are also shown (open purple triangles; N=7). The shaded areas are 1σ, and 'N' refers to the number of days analyzed.

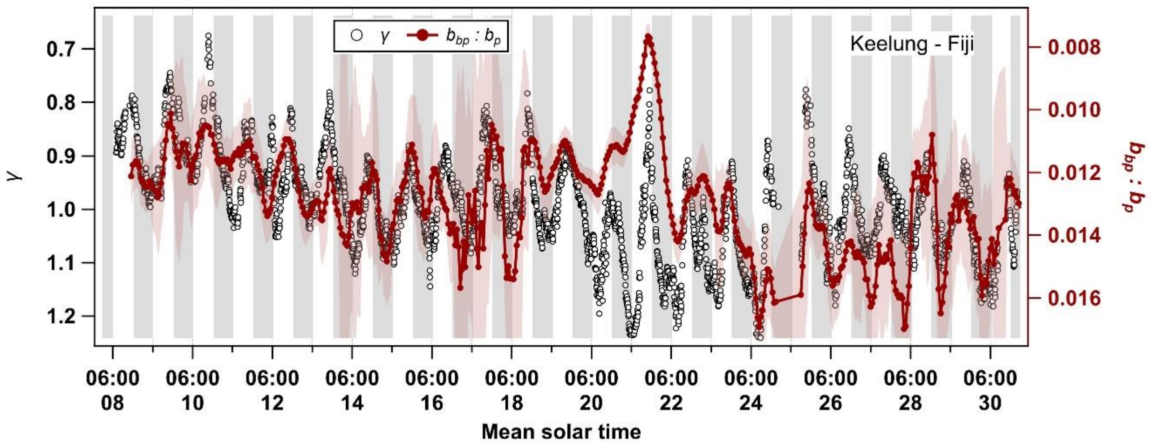

Supplementary Figure 14. Contribution of small (~<1 µm) particles to γ variations. Particle size index (*γ*, black circles) and backscattering to total particulate scattering ratio ($b_{bp} : b_p$) for the Keelung – Fiji leg Vertical shaded areas indicate nighttime.

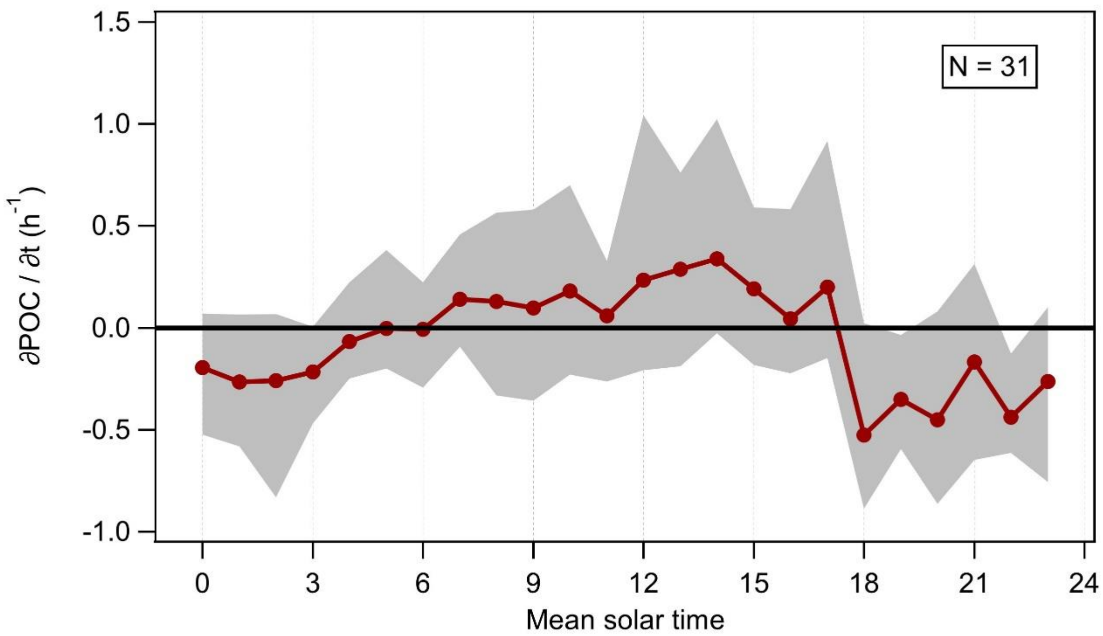

Supplementary Figure 15. Diurnal anomaly of the rate of change of particulate organic carbon (POC) at a depth of around 0.5 – 3.0 m during the Keelung – Fiji leg. The baseline was taken to be the mean value between midnight and five in the morning. The shaded areas are 1σ. 'N' refers to the number of days analyzed.

Supplementary Table 1.
SEM-EDX analysis filter times, latitude, longitude and number of particles analyzed.

**Filter type***	**Initial Solar time****	**Final solar time**	**Latitude range**	**Longitude range**	**# Particles counted**	**Sea salt fraction**
N	03-May 20:58:40	04-May 08:55:28	21 - 20.5	128.2 – 129.5	233	0.66
D	04-May 09:08:16	04-May 20:48:00	20.5 – 20.3	129.5 – 130.4	781	0.46
N	04-May 21:05:04	05-May 08:02:08	20.3 – 19.9	130.4 – 131.5	276	0.66
D	05-May 08:27:44	05-May 20:07:28	19.9 – 19.6	131.5 – 132.6	254	0.72
N	05-May 20:16:00	06-May 08:55:28	19.6 – 19.5	132.6 – 134	388	0.51
D	06-May 09:04:00	06-May 21:17:52	19.5 – 18.6	134 – 134.6	438	0.45
N	06-May 21:26:24	07-May 09:40:16	18.6 – 17.7	134.6 – 135.5	342	0.72
D	07-May 09:53:04	07-May 20:54:24	17.7 – 16.7	135.5 – 135.8	211	0.75
N	07-May 21:02:56	08-May 08:21:20	16.7 – 15.9	135.8 – 136.8	257	0.66
D	08-May 08:29:52	08-May 20:48:00	15.9 – 14.7	136.8 – 137.1	502	0.48
N	08-May20:56:32	09-May 08:19:12	14.7 – 13.8	137.1 – 137.8	95	0.57
D	09-May 08:27:44	09-May 20:41:36	13.8 -12.6	137.8 – 138.5	260	0.81
N	09-May 20:54:24	10-May 08:34:08	12.6 – 11.4	138.5 – 139.2	298	0.93
D	10-May 08:46:56	10-May 20:48:00	11.4 – 10.3	139.2 – 140	411	0.91
N	10-May 21:00:48	11-May 09:06:08	10.3 – 9	140 – 140.5	325	0.91
D	11-May 09:10:24	11-May 21:20:00	9 – 7.9	140.5 – 140.9	416	0.89
N	11-May 21:32:48	12-May 09:21:04	7.9 – 6.8	140.9 – 141.5	253	0.93
D	12-May 09:29:36	12-May 20:26:40	6.8 – 6	141.5 – 142.3	201	0.88
N	12-May 20:43:44	13-May 08:14:56	6 – 5.3	142.3 – 143.4	137	0.91
D	13-May 08:23:28	13-May 20:28:48	5.3 – 4.7	143.4 – 144.5	338	0.83
N	13-May 20:37:20	14-May 09:08:16	4.7 – 4.3	144.5 – 145.6	317	0.81
D	14-May 09:12:32	14-May 20:22:24	4.3 -4.1	145.6 – 146.7	213	0.94
N	14-May 20:35:12	15-May 08:06:24	4.1 – 3.6	146.7 – 147.6	107	0.95
D	15-May 08:10:40	15-May 20:33:04	3.6 – 3.2	147.6 – 148.8	113	0.86
N	15-May 20:41:36	16-May 08:29:52	3.2 – 2.9	148.8 – 149.8	80	0.97
D	16-May 08:34:08	16-May 20:35:12	2.9 – 2.3	149.8 – 150.8	190	0.99
N	16-May 20:43:44	17-May 08:49:04	2.3 – 1.7	150.8 – 151.7	291	0.89
D	17-May 08:53:20	17-May 20:24:32	1.7 – 1.3	151.7 – 152.4	232	0.98
N	17-May 20:33:04	18-May 08:25:36	1.3 - 1	152.4 – 153.2	307	0.91

* The letter marked in bold and Italic are shown in Fig. 1B. **All filter were collected in the year 2017

Supplementary Table 2.
***Tara's* Meteorological data**

Parameter	Sensor	Measurement	Precision	Resolution
Pressure	Vaisala PTU200	Every 5 s<br>Median over 3 minutes	0.1 hPa	0.1 hPa
Temperature	Vaisala HMP45D	Every 5 s<br>Median over the minute	0.1 °C	0.1 °C
Humidity	Vaisala HMP45D	Every 5 s<br>Median over the minute	3%	1%
Wind	Gill Windsonic WS2	Sensor sending frames every 250 ms<br>1-minute average true wind computed	Speed 2% at 12 m/s<br>Direction 3° at 12 m/s	Speed : 0.01 m/s<br>Direction 0.1°

Supplementary References

1. Flores, J. M. *et al.* Tara Pacific Expedition's Atmospheric Measurements of Marine Aerosols across the Atlantic and Pacific Oceans: Overview and Preliminary Results. *Bulletin of the American Meteorological Society* **101,** E536–E554 (2020).

2. Flores, J. M., Trainic, M., Borrmann, S. & Rudich, Y. Effective broadband refractive index retrieval by a white light optical particle counter. *Phys. Chem. Chem. Phys.* **11,** 7943–7950 (2009).
