# Supplementary material for: Diel cycle of sea spray aerosol concentration
Source: Nat Commun. 2021 Sep 16;12:5476. doi: 10.1038/s41467-021-25579-3 (PMC8445914; doi:10.1038/s41467-021-25579-3)
Supplement: Supplementary file 3 — Reporting Summary [file 41467_2021_25579_MOESM3_ESM.pdf]

nature research

Corresponding author(s): J. Michel Flores

Last updated by author(s): Jul 18, 2021

Reporting Summary

Nature Research wishes to improve the reproducibility of the work that we publish. This form provides structure for consistency and transparency in reporting. For further information on Nature Research policies, see our Editorial Policies and the Editorial Policy Checklist.

Statistics

For all statistical analyses, confirm that the following items are present in the figure legend, table legend, main text, or Methods section.

n/a	Confirmed	
☐	☒	The exact sample size (*n*) for each experimental group/condition, given as a discrete number and unit of measurement
☐	☒	A statement on whether measurements were taken from distinct samples or whether the same sample was measured repeatedly
☐	☒	The statistical test(s) used AND whether they are one- or two-sided<br>*Only common tests should be described solely by name; describe more complex techniques in the Methods section.*
☒	☐	A description of all covariates tested
☒	☐	A description of any assumptions or corrections, such as tests of normality and adjustment for multiple comparisons
☐	☒	A full description of the statistical parameters including central tendency (e.g. means) or other basic estimates (e.g. regression coefficient) AND variation (e.g. standard deviation) or associated estimates of uncertainty (e.g. confidence intervals)
☐	☒	For null hypothesis testing, the test statistic (e.g. *F*, *t*, *r*) with confidence intervals, effect sizes, degrees of freedom and *P* value noted<br>*Give P values as exact values whenever suitable.*
☒	☐	For Bayesian analysis, information on the choice of priors and Markov chain Monte Carlo settings
☒	☐	For hierarchical and complex designs, identification of the appropriate level for tests and full reporting of outcomes
☐	☒	Estimates of effect sizes (e.g. Cohen's *d*, Pearson's *r*), indicating how they were calculated

Our web collection on statistics for biologists contains articles on many of the points above.

Software and code

Policy information about availability of computer code

Data collection	No software was used
Data analysis	The codes were written in the sortware program IGOR Pro 7.08 . They were written only for the analysis of the data presented. The Bruker ESPRIT 2.1.217832 feature software package for automatic particle detection and chemical classification in EDS was used, in a Zeiss Sigma500 SEM with a Bruker XFlash®-6|60 Quantax EDS detector.

For manuscripts utilizing custom algorithms or software that are central to the research but not yet described in published literature, software must be made available to editors and reviewers. We strongly encourage code deposition in a community repository (e.g. GitHub). See the Nature Research guidelines for submitting code & software for further information.

Data

Policy information about availability of data

All manuscripts must include a data availability statement. This statement should provide the following information, where applicable:

- Accession codes, unique identifiers, or web links for publicly available datasets
- A list of figures that have associated raw data
- A description of any restrictions on data availability

All data for this article have been deposited in the open access Weizmann Institute’s institutional repository and are available in this link: doi.org/10.34933/wis.000392.

Field-specific reporting

Please select the one below that is the best fit for your research. If you are not sure, read the appropriate sections before making your selection.

☐ Life sciences ☐ Behavioural & social sciences ☒ Ecological, evolutionary & environmental sciences

For a reference copy of the document with all sections, see nature.com/documents/nr-reporting-summary-flat.pdf

Ecological, evolutionary & environmental sciences study design

All studies must disclose on these points even when the disclosure is negative.

Study description	Measurements of marine aerosol size distributions across the Atlantic Ocean, the Caribbean Sea, and the Pacific Ocean, along side meteorological data, surface ocean physical properties and approximate size of marine particles.
Research sample	The atmospheric marine boundary layer and the ocean surface
Sampling strategy	We took the raw counts (per minute) measured by the particle counter. The raw counts can be assumed to follow a Poisson distribution, therefore their standard deviation, $\sigma=\sqrt{\mu}$, where μ is the mean. We sampled for 282 days, and therfore had over 1,000,000 sample points. The sample points were devided then into 24hr periods. We belive this amount of data is sufficient to draw conclusions as normally sea expeditions last about 30 days or less.<br>For the aerosol filters we sampled for periods of about 12 hours with a flow of 30 lpm. We decided to sample for 12 hour periods to try to differenciate between night and day.<br>The AC-S data recorded data every minute. The instrument was turned off if the algae conceantrtion was too high (e.g., near Japan).
Data collection	Data was collected with an optical particle counter (OPC; EDM-180 GRIMM Aerosol Technik Ainring GmbH & Co. KG, Ainring, Germany), for continuous aerosol size distribution measurements (from 0.25 – 32 µm, sorted into 31 bins), and a custom-made aerosol filter system consisting of four 47mm filter holders and one vacuum pump (Diaphragm pump ME 16 NT, VACUUBRAND BmbH & Co KG, Wertheim, Germany) were installed aboard R/V Tara. Two separate inlets, located next to each other, were constructed out of conductive tubing of 1.9 cm inner diameter and a funnel (allowing the collection of all diameters) and mounted on the rear backstay of Tara. For the Atlantic Ocean measurements, from Lorient, France to Miami, U.S.A., the inlet was installed half way up the backstay (~15m asl) and after Miami, the inlet was relocated to the top of the backstay (~27m asl).The instruments were installed in the sail boat and left running independently, with researchers and crew members checking them continuously. The collected filters were changed every 12 hours by J. Michel Flores. The meteorological data was collected with the weather station on board Tara and operated by Meteo France (Station Bathos II, Météo France).<br>For the water measurements, the inline system consisted of a Sea-Bird Electronics SBE45 MicroTSG for measurements of sea surface temperature (SST) and salinity and an AC-S spectrophotometer (WET Labs, Inc.) measuring hyperspectral particulate absorption (ap) and particulate attenuation (cp) with a ~4 nm resolution, and an ECO-BB3 (WetLabs Inc.) set in a BB-box of ~4.5 L measuring particulate backscattering at three wavelength (470 nm, 532 nm and 650 nm), altogether mounted in an autonomous setup.<br>Pictures of the ocean-atmosphere state were taken with a GoPro camera installed at portside; the GoPro took one picutre every minute.<br>Long rains periods were anotated in the log sheet with permanent markers.
Timing and spatial scale	The expedition began on 28 May 2016 from Lorient, France, and finished the first year of the campaign on June 17, 2017, in Whangarei, New Zealand. The expedition was design to sample coral reefs, the island stop overs were design for the coral reef research. The aerosol instrumentation ran, for the most part, continuously regardless of the stop over.
Data exclusions	The data was checked for pollution periods, all periods recognized as pollution (e.g., by engine exhaust), were excluded from the analysis.
Reproducibility	Given it is filed data, in order for another group to be able to reproduce our findings, we clearly marked the route and dates of the expedition and the instrumentation as well. We calibrated the optical particle counter before the expedition and in the Island of Guam about 8 months into the expedition. We believe that with this information the measurements can be reproduced by any other research vessel.
Randomization	Randomization of the optical particle counter data and the AC-S is not relevant for this study. We did continuous measurements in the marine boundary layer and the ocean surface where several scientist and crew members were involved to check the optical particle counter and the AC-S were running without errors. For the aerosol filters all scientist and crew members followedg a specific protocol to change the filters. The initial and final times (in UTC) of sampling were written in a log sheet and a and a random barcode was given to it. To analyze the aerosol filters in the SEM, we first identified the two week period to be analyzed, then the filters were analyzed by randomly taken a filter, which were labeled with different barcodes, after the analysis of the filters were done, they were assingned to the appropriate date written in the log sheets.
Blinding	Not relevant, we performed continuous measurements of marine aerosols and the surface of the ocean. For the aerosol filter measurements, a protocol was written for any researcher on board or crew member to follow. This involved labeling the collected filter and a log sheet where initial and final times, in UTC, were written for each filter. Later, to analyze the filters the log sheets were used to find the relevant dates. We also did a handling blank every few days.

Did the study involve field work? ☒ Yes ☐ No

Field work, collection and transport

Field conditions	The field conditions varied throughout the expedition. The air temperature varied from 5 - 32 °C, the relative humidity from 17 - 99%. The sea surface temperature varied from 10 to 32°C. Long rain events were recorded by a scientist or a crew member. In the tropics most rain events were sporadic and short lived, less than 10 min.
Location	The expedition took place in the Atlentic Ocean, Caribbean Sea, and Pacific Ocean. It started at 47.7 N, 3.4W and directed south west. It passed through Miami, U.S.A, then went around the cariabbean sea to go to the Panama canal. After crossing the Panama canal the boat went to Easter Island, and from there it went northwest till it reached Japan, where it reached 35.4 N 139.7 E. After this the boat went to Taiwan (25.1 N, 121.7 E). After the Taiwan stop over, the boat went southeast towards Fiji (18.3 S 178 E), and finally it went south to New Zealand, finishing at 35.8 S 174.5 E.<br>Aerosols were measured at ~15m above sea level in the Atlantic and ~30m above sea level for the rest of the expedition. And ocean particles were measured at a depth of 0.5-3m depth, depending on the sea state.
Access & import/export	Permits to access the areas sampled were given by: Ministerio de Ambiente, Republica de Panama on 13-06-2016, No. SE/AP-18-16; Parques Nacionales Naturales de Colombia on 22-02-2016, Codigo: AMSPNN_FO_16, No: 2016230420500002E; Armada de Chile Servicio Hidrografico y Oceanografico on 29-08-2016, No. 13270/24/457/Vrs.; Convention sur le commerce international des espèces de flore et de faune sauvages menacées d'extinction, Polynésie Française (France), on 03/11/2016 for No. FR1698700198-E and on 21/11/2016 for No. FR16987002189-E; Cook Island Research Committee, Cook Islands, on 12/09/2016, file ref: 510.3; Ministry of Natural Resources and Environment, Samoa, on 29/11/2016, No. SAMC16012; Administration superieure des Iles Wallis et Futuna, Terriroire des Iles Wallis et Futuna, on 24/11/2016, No. 2016-527; Ministry off Foreign Affairs, Trade, Tourism, Environment and Labour, Government of Tuvalu, on 19/10/2016, No. 2016/753527; Environment and Conservation Division, Republic of Kiribati, on 24/11/2016, No. 015/16; Department of Resources & Development, Federated States of Micronesia, on 19/01/17, No. CFM17-01-01; Department of Agriculture, Guam, on 4/2/2017, No. SC-17-004; Ministry of Agriculture, Forestry and Fisheries, Japan, on 18/1/2017, No. 019; Ministry of Foreign Affairs, Republic of Fiji, on 11/07/2017, No. 456/2017.
Disturbance	We believe we did not cause any disturbance

Reporting for specific materials, systems and methods

We require information from authors about some types of materials, experimental systems and methods used in many studies. Here, indicate whether each material, system or method listed is relevant to your study. If you are not sure if a list item applies to your research, read the appropriate section before selecting a response.

Materials & experimental systems

n/a	Involved in the study	
☒	☐	Antibodies
☒	☐	Eukaryotic cell lines
☒	☐	Palaeontology and archaeology
☒	☐	Animals and other organisms
☒	☐	Human research participants
☒	☐	Clinical data
☒	☐	Dual use research of concern

Methods

n/a	Involved in the study	
☒	☐	ChIP-seq
☒	☐	Flow cytometry
☒	☐	MRI-based neuroimaging
